# Predictive value of glucose transporter-1 and glucose transporter-3 for survival of cancer patients: A meta-analysis

**DOI:** 10.18632/oncotarget.14570

**Published:** 2017-01-10

**Authors:** Xiu Chen, Peng Lu, Siying Zhou, Lei Zhang, Jian-hua Zhao, Jin-hai Tang

**Affiliations:** ^1^ The First Clinical School of Nanjing University of Chinese Medicine, Nanjing, China; ^2^ Department of General Surgery, Jiangsu Cancer Hospital Affiliated to Nanjing Medical University, First Affiliated Hospital of Nanjing Medical University, Nanjing, China; ^3^ School of Public Health Nanjing Medical University, Nanjing, China; ^4^ Xuzhou Medical University, Xuzhou, China; ^5^ Center of Clinical Laboratory Science, Jiangsu Cancer Hospital Affiliated to Nanjing Medical University, Nanjing, China

**Keywords:** glucose transporter, GLUT, survival, cancer, meta-analysis

## Abstract

**Background and Objective:**

The role of glucose transporters in cancers remains contradictory. We conducted a systematic review and meta-analysis to assess the association between overall survival and glucose transporter s (GLUTs) 1 and 3 to find an accurate prognostic biomarker.

**Methods:**

We systematically searched the PubMed, EMbase and Medline databases for relevant published studies that were consistent with the eligible criteria up to January 2016, and calculated pooled estimated hazard ratios of GLUT-1 and -3′s expressions in different cancer types and ethnic populations. Random-effects models were used to assess estimates from studies with significant heterogeneities.

**Results:**

Overall, 12 studies concerning GLUT 1 and 2 studies concerning GLUT 3, which involved 2008 participants when combined, were included in this analysis. We found that overexpression of GLUTs were significantly correlated to poorer survival rates (HR=1.63, 95%CI=1.09-2.44 and HR=1.89, 95%CI=1.28-2.81). In the subgroup analysis, the GLUT 1 up-regulation was correlated with negative overall survival in pancreatic cancer and gastric cancer and with better overall survival in colorectal cancer. In addition, overexpression of GLUT 1 was associated with a poorer prognosis in the Asian population, while no significance was found in the non-Asian subgroup. However, limitations do exist, which could be handled better.

**Conclusions:**

A combination of GLUTs 1 and 3 might help predict malignancy of cancers and direct effective cancer therapy.

## INTRODUCTION

Cancers remain to be a heavy burden to human health and survival, with estimated 14.1 million new cases (ranking as lung, breast and colorectal cancer) and 8.2 million deaths (lung cancer, liver cancer and stomach cancer on the top) in 2012 worldwidely [1]. Regardless of the considerable efforts in interpreting carcinogenic mechanisms and in developing the advanced diagnostic approaches as well as treatments, the incidence and mortality rates of several cancers still increased dramatically over time [2].

Warburg first showed in the 1920s that increased glucose metabolism in cancer cells were in need of plenty of energies [3]. The glucose metabolism of cells depends on the transportation of membrane transport proteins namely the glucose transporter (Glut) family [4].

To date, numerous evidences have reported that deregulated expressions of GLUT1 and GLUT 3 are associated with malignancy of several cancer types [5] including pancreatic [6, 7], gastric [8, 9], colorectal [10, 11], uterine [12, 13], oral [14, 15], neuroblastic cancer [16] and malignant peritoneal mesothelioma [17], however, some studies depicted longer survival associated with GLUTs expression while others detected no correlation or poorer survival. Meanwhile, no meta-analysis have been conducted to investigate the association of GLUT-1 with GLUT-3 and the survival of cancer patients. Also, no evidence had evaluated their correlation in ethnic subgroups. Therefore, a meta-analysis including all available studies (2008 participants) was implemented to assess the relationship between GLUTs expressions and the overall survival of cancer patients. Consequently, the result would be of great importance in the enhancement of prediction and management of cancer patients.

## RESULTS

### Study characteristics

**Figure 1:**
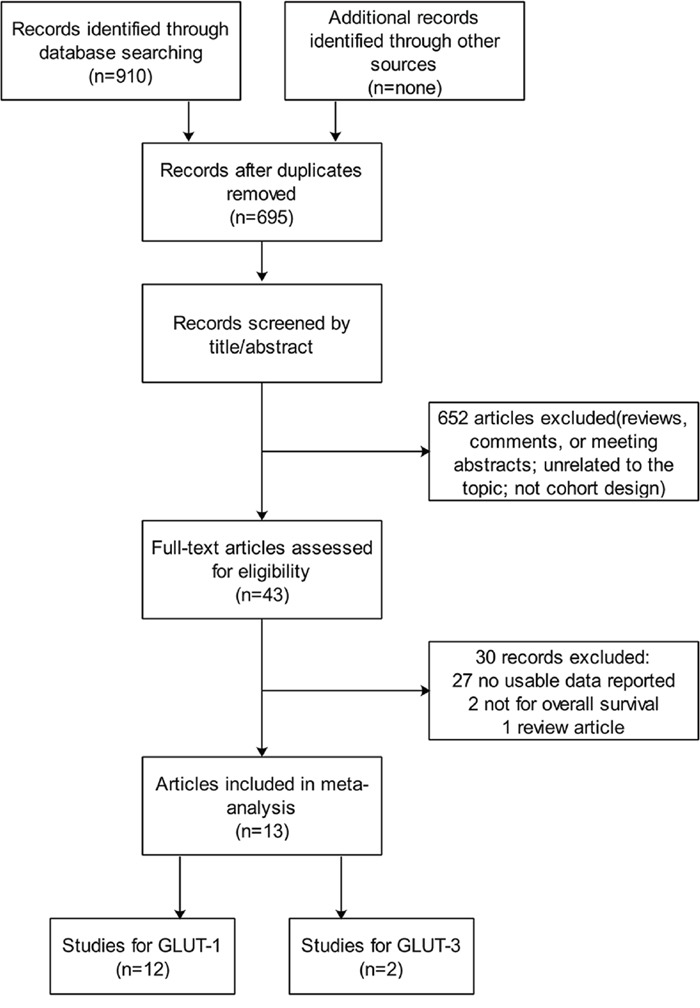
Flow chart of the literature search and selection strategy

The search and selection processes are described in Figure 1. A total of 910 studies were found through retrieval, of which 215 were duplicates. Of the remaining 695 studies, 652were excluded for either the reviews, comments, or abstracts, being unrelated to the topic and/or not of cohort design. Consequently, after further excluding 30 additional studies for various reasons, 13 studies were available as full texts for the final analysis. Among those 13 studies, 12, which included a total of 1,716 participants, compared GLUT-1 and OS, and 2, which included 292 participants, compared GLUT-3 and OS. All studies were representative, and a total of 2008 participants took part in the present analysis (Table 1).

**Table 1:** Baseline of eligible studies of GLUT-1

Author	Year	Country	Ethnicity	Cases	Age (years)	male %	Diagnostic methods	High expression	Median follow-up time (months)	Tumor type
Min Yu	2015	China	Chinese	106	60.42	53.8	IHC, qRT-PCR	<30%	21.9	pancreatic cancer
Sara	2009	Italy	Italian	60	67.3	50	IHC	<50%	13.5	pancreatic cancer
Tetsuo	2001	Japan	Japanese	617	60.5	60.6	IHC	<30%	N/A	gastric cancer
Hans	2015	Germany	Caucasion	150	64	76	IHC	<10%	33.2	gastric cancer
Arjen	2007	Netherlands	Caucasion	133	69	41	IHC	<50%	N/A	colorectal cancer
Elena	2012	Italy	Italian	135	68	46.7	IHC	<50%	35	colorectal cancer
Pawel	2014	Poland	Caucasion	92	65.1	N/A	IHC	N/A	N/A	endometrial cancer
Xin-Qiong Huang	2014	China	Chinese	132	51	N/A	IHC	<75%	45	cervical aquamous cell carcinoma
Martin	2002	Germany	Caucasion	118	58	N/A	IHC, PET	<50%	74	oral squamous cell carcinoma
Yusuke	2006	Japan	Japanese	49	55	51	IHC, qRT-PCR	<15%	N/A	malignant salivary gland tumor
J. Hommell-Fontaine	2013	UK	Caucasion	28	54	64	TMA, IHC	<5%	34	malignant peritoneal mesothelioma
Pramila	2013	UK	Caucasion	96	21.66 months	N/A	IHC	N/A	86	neuroblastic tumour
**Baseline of eligible studies of GLUT-3**										
**Author**	**Year**	**Country**	**Ethnicity**	**Cases**	**Age**	**male %**	**Diagnostic methods**	**High expression**	**Median follow-up time (months)**	**Tumor type**
Fernanda	2010	Brazil	Caucasion	142	57	78.9	TMA, IHC	<10%	64.9	oral squamous cell carcinoma
Hans	2015	Germany	Caucasion	150	64	76	IHC	<10%	33.2	gastric cancer

IHC: Immunohistochemistry; TMA: Tissue microarray construction.

### GLUT-1 expression and overall survival

**Figure 2:**
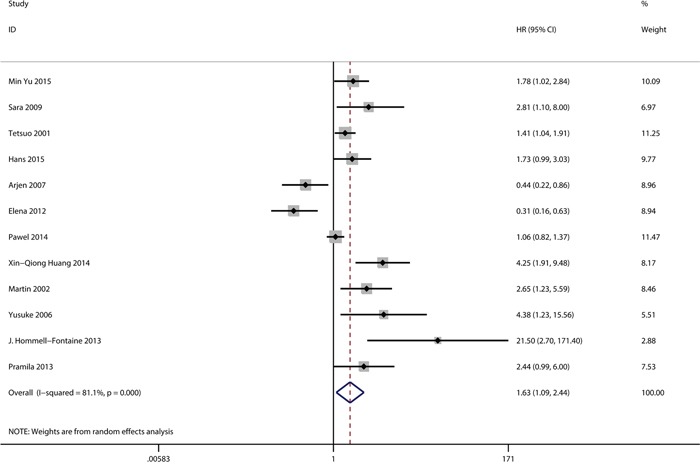
Meta-analysis with a random-effect model for the association between GLUT-1 and OS

**Figure 3:**
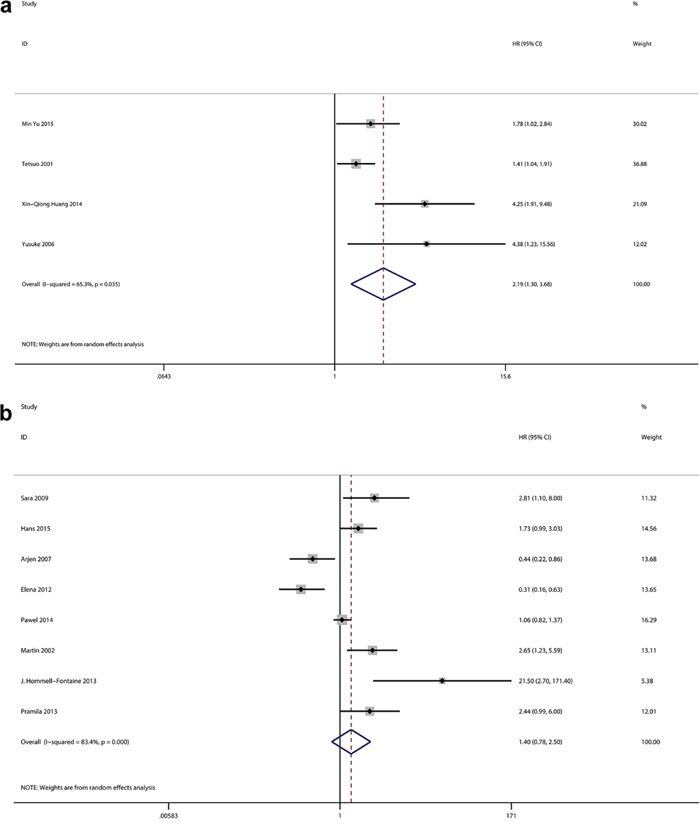
**a**. Meta-analysis with a random-effect model for the association between GLUT-1 and OS in Asian population. **b**. Meta-analysis with a random-effect model for the association between GLUT-1 and OS in non-Asian population.

**Figure 4:**
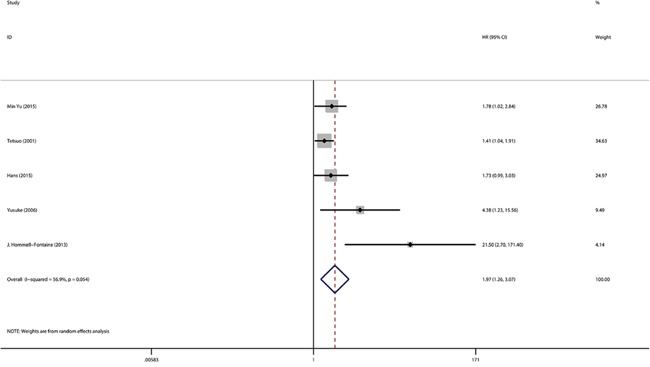
Meta-analysis with a random-effect model for the association between GLUT-1 and OS in percentage of high GLUT-1 expression <30%

The expression of GLUT-1 and OS were reported in 12 studies that included 1716 patients. From the overall analysis, a greater than 1.6-fold higher risk of a poor prognosis was observed for a GLUT-1 positive expression compared with a GLUT-1 negative expression(Figure 2). The analysis indicated that GLUT-1 expression was potentially relevant to a poor prognosis (HR=1.63, 95%CI=1.09-2.44, heterogeneity p <0.005). Out of the 12 studies, 2were conducted on pancreatic cancer, 2 were on gastric cancer and 2were on colorectal cancer. In the subgroup analysis, the GLUT-1 positive expression was significantly correlated with the poor outcome of pancreatic cancer (HR=1.96, 95%CI=1.24-3.09, heterogeneity p=0.423) (Supplementary Figure 1) and gastric cancer (HR=1.48, 95%CI=1.13-1.93, heterogeneity p=0.529) (Supplementary Figure 2), while an evidently positive relation was detected in colorectal cancer patients (HR=0.37, 95%CI=0.23-0.60) (Supplementary Figure 3). Furthermore, the subgroup analysis suggested the GLUT-1 led to an increased risk of mortality in the Asian population(Figure 3a), however, no significance was discovered in the association between GLUT-1 and the non-Asian population(Figure 3b) (see detailed data in Table 2). When analyzing the studies with <30% positive GLUT-1 expression independently, we concluded that GLUT-1 still predicted negative prognoses in cancers (HR=1.97, 95% CI: 1.26–3.07, heterogeneity p=0.054) (Figure 4).

**Table 2:** GLUT-1, -3 and OS

GLUT-1				GLUT-3		
GLUTs	Studies	HR (95%CI)	P	Studies	HR (95%CI)	P
Overall	12	1.63 (1.09-2.44)	0.018	2	1.89 (1.28-2.81)	0.002
Pancreatic	2	1.96 (1.24-3.09)	0.004	-		
Gastric	2	1.48 (1.13-1.93)	0.004	-		
Colorectal	2	0.37 (0.23-0.60)	0.000	-		
Asian	4	2.19 (1.30-3.68)	0.003	-		
Non-Asian	8	1.40 (0.78-2.50)	0.261	-		

HR: Hazard ratio; CI: Confidence intervals.

### GLUT-3 expression and overall survival

**Figure 5:**
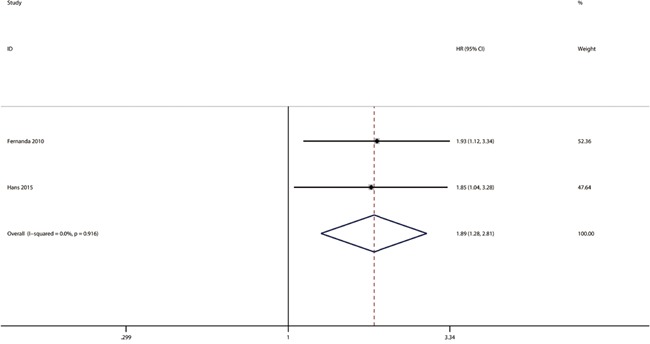
Meta-analysis with a random-effect model for the association between GLUT-3 and OS

Two studies were available for the analysis of GLUT-3 expression and OS in this meta-analysis(Figure 5, Table 2). The pooled HR from the 2 investigations was 1.89 with a 95% CI ranging from 1.28 to 2.81(heterogeneity p=0.916). No publication bias was found.

### Publication bias

A funnel plot was used to evaluate possible publication bias. Priority in positive research results was found in articles concerning GLUT-1 expression and more studies were needed to alleviate publication bias.

### Sensitivity analysis

We performed a sensitivity analysis to evaluate the reliability of the results. Every study was removed in sequence at the same time. The results indicated that five literatures may have affected the reliability because of a negative result [10, 11], short follow-up time [9], and large [8] or small [12] sample numbers(Table 3). However, the conclusions derived from our meta-analysis are relatively credible.

**Table 3:** Sensitivity analysis results

Studies	HR (95%CI)
Min Yu 2015	1.63 (1.65,2.60)
Sara 2009	1.57 (1.03,2.39)
Tetsuo 2001	1.72 (1.05,2.80)
Hans 2015	1.64 (1.05,2.55)
Arjen 2007	1.83 (1.23,2.73)
Elena 2012	1.86 (1.28,2.69)
Pawel 2014	1.77 (1.09,2.89)
Xin-Qiong Huang 2014	1.48 (0.99,2.22)
Martin 2002	1.56 (1.02,2.38)
Yusuke 2006	1.54 (1.02,2.32)
J. Hommell-Fontaine 2013	1.50 (1.02,2.22)
Pramila 2013	1.58 (1.04,2.42)

HR: Hazard ratio; CI: Confidence intervals.

## DISCUSSION

Cancer mortality seems to elevate at a stable rate; therefore, it requires efficient diagnostic and therapeutic approaches [1]. Increased glucose uptake is an acknowledged characteristic of cancer cells, and applying this feature when imaging tumors using radioactive tomography has become a diagnostic standard [18, 19]. However, no definite evidence distinguishes different types of glucose transporters that affect the glucose uptake from one another.

Expression of GLUTs 1 and 3 could be induced simultaneously by the hypoxia inducible factor 1 [20]. Specifically, GLUT-1 was reported to correlate with the poor prognosis of diverse cancer types, such as colorectal cancer [21], while Arjen [10] and Elena [11] et al. demonstrated that GLUT-1 improved the outcomes of colorectal cancer patients (although their findings were inconsistent). Additionally, no studies have delivered the survival rate of cancer patients with GLUT-1 expression. Therefore, we carried out this meta-analysis to analyze the association between patients' outcomes and GLUTs-1 with -3 expressions, both overall and within ethnic groups.

In the present research, 12 studies concerning GLUT-1 and 2 studies concerning GLUT-3, which included 2008 participants between them, were included. We demonstrated that GLUT-1 expression was significantly correlated with lower overall survival rate in the studied cancers. Furthermore, in the sub-group analysis, expression of GLUT-1 was associated with shorter OS in pancreatic cancer, gastric cancer and Asian populations, while colorectal cancer patients showed a positive relation to GLUT-1. We also found a correlation between GLUT-3 and the negative survival, mainly in oral squamous cell cancer. A funnel plot was used to evaluate publication bias. Priority was found in positive research results from the articles concerning GLUT-1 expression; however, more studies were needed to alleviate publication bias. When calculating the effect of GLUT-1 on survival, publication bias appeared. We were unable to avoid that aspect because positive results were preferred by journals.

Limitations in this study should be acknowledged. Firstly, the number of subgroup studies was too small to calculate an accurate estimation of the relationship between the GLUT and OS rates. Secondly, because meta-analysis is based on published literature, a significant amount of individual data was not available. Thirdly, when attempting to detect OS in different regions, we were unable to distinguish between longer OS and shorter OS, which may have contributed to publication bias. Two [13, 17] of the studies used univariate analysis to assess the relationship between GLUT-1 and OS; however, they ignored other potential influencing factors. The others were all conducted via multivariate analysis to define the independent prognostic role of GLUT-1.

In conclusion, in this meta-analysis, we identified the discriminated role of GLUTs-1 and -3 in malignancies, and emphasized the predictive function of combing GLUT-1 with GLUT-3 in cancer prognoses. The results provided a novel rationale for applying GLUTs to help predict effective cancer therapies.

## MATERIALS AND METHODS

### Information source and search strategy

In order to identify relevant studies, a systematic search was performed in PubMed, Medline and Embase up to January 2016. The following terms were applied to search for relevant researches in the databases: “glucose transporter 1” or “GLUT-1” and “glucose transporter 3” or “GLUT-3” and “cancer” in combination with “prognosis”. In case of ignoring any important and useful information, we also screened the reference lists of key studies and reviews.

### Inclusion and exclusion criteria

Researches retrieved from the databases were first scanned through titles with abstracts and then full-text studies were further reviewed for eligibility. Eligible studies were selected in accordance with the following inclusion criteria: human-based studies; pathologically confirmed cancer with immunohistochemistry detection; full text written in English; evaluation of the correlation between GLUT-1 and -3 expressions and overall survival (OS).

If the studies met the following selection criteria, they would be excluded: case reports, editorials and animal studies; systematic review and meta-analysis; studies described the association of GLUT expression levels and survival days; the hazard ratio (HR) and 95% confidence interval (95% CI) did not reported or could not be calculated; full text could not be found; full texts were not published in English.

Two investigators (XC and PL) participated in the search of available references individually, differences were resolved by discussion with another author (JZ) and they reached the consensus on each eligible study.

### Data extraction

The following data were collected: first author, publishing year, country of origin, numbers of cases, age, median of survival time, percent of male, diagnostic methods of GLUT expression, high expression, median follow-up time and tumor type.

### Statistical analysis

The meta-analysis was performed using STATA 13.1 software. The HR and 95%CI were used to assess the relationship between GLUT-1, GLUT-3 expression and survival of cancers. We used χ^2^ and I-squared (I^2^) to evaluate the heterogeneity. The random-effect model was adopted if the p≤0.10 and I^2^≥50%, which meant existing heterogeneity among studies model. Otherwise, the fixed-effect model was applied. To assess the covariate effects, we classified ethnic subgroups as Asians and non-Asians. Funnel plots was used to assess the publication bias. Two-sided p <0.05 was considered statistically significant. Reliability of the conclusion was obtained by sensitivity analysis.

## SUPPLEMENTARY FIGURES


