# Supplementary material for: Predictive value of glucose transporter-1 and glucose transporter-3 for survival of cancer patients: A meta-analysis
Source: Oncotarget. 2017 Jan 10;8(8):13206–13. doi: 10.18632/oncotarget.14570 (PMC5355089; doi:10.18632/oncotarget.14570)
Supplement: Supplementary file 1 [file oncotarget-08-13206-s001.pdf]

Predictive value of glucose transporter-1 and glucose transporter-3 for survival of cancer patients: A meta-analysis

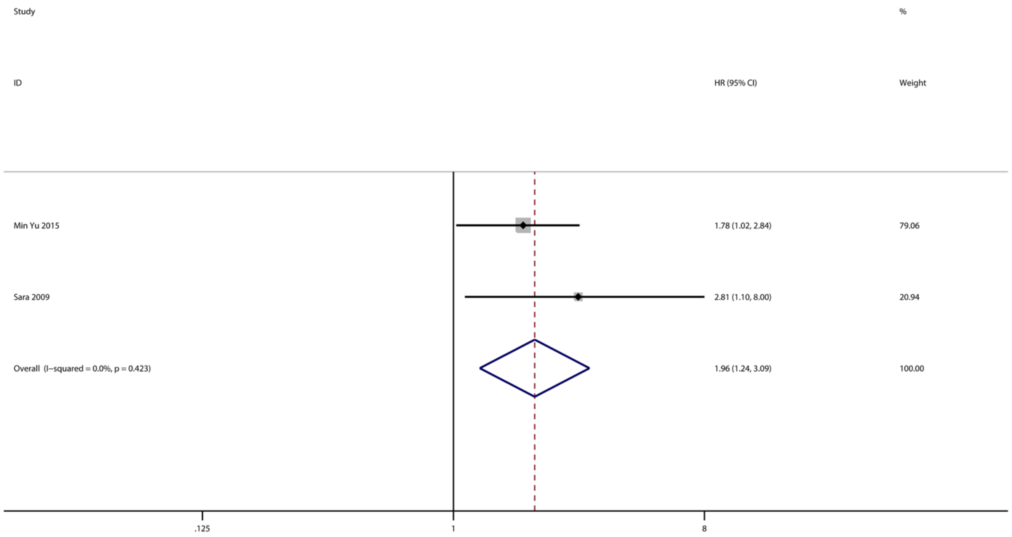

Supplementary Figure 1: Meta-analysis with a random-effect model for the association between GLUT-1 and OS in pancreatic cancer.

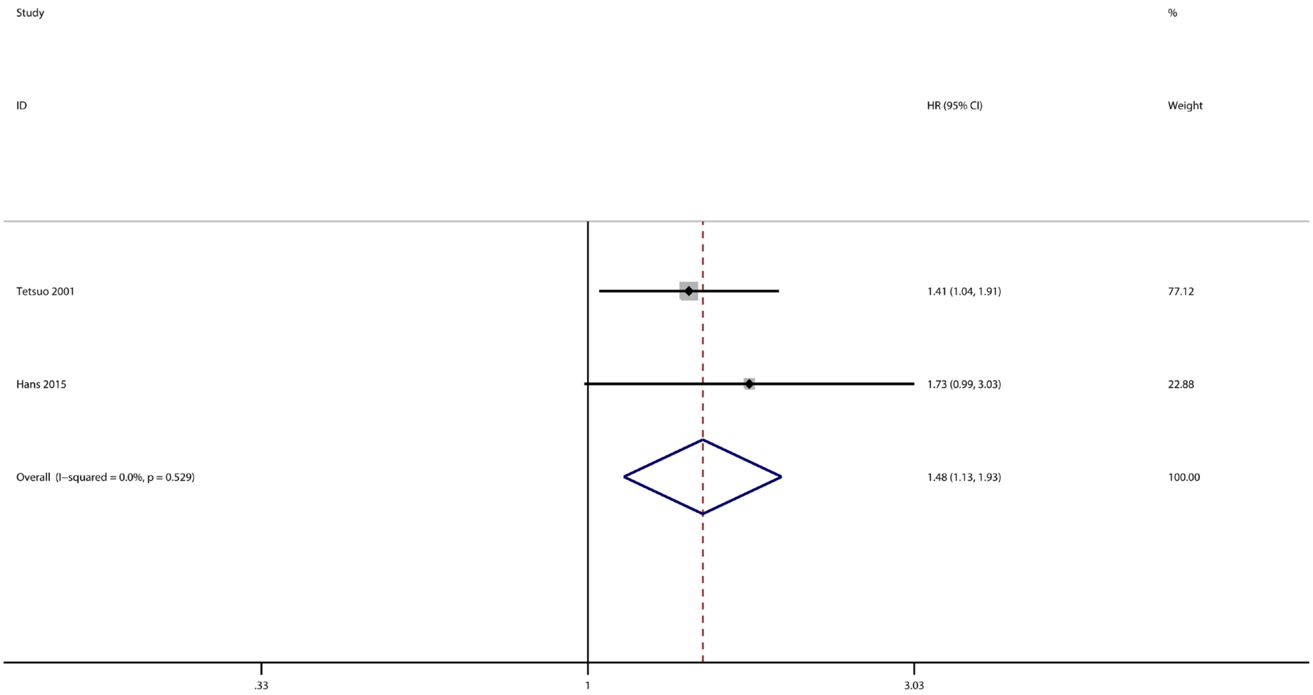

Supplementary Figure 2: Meta-analysis with a random-effect model for the association between GLUT-1 and OS in gastric cancer.

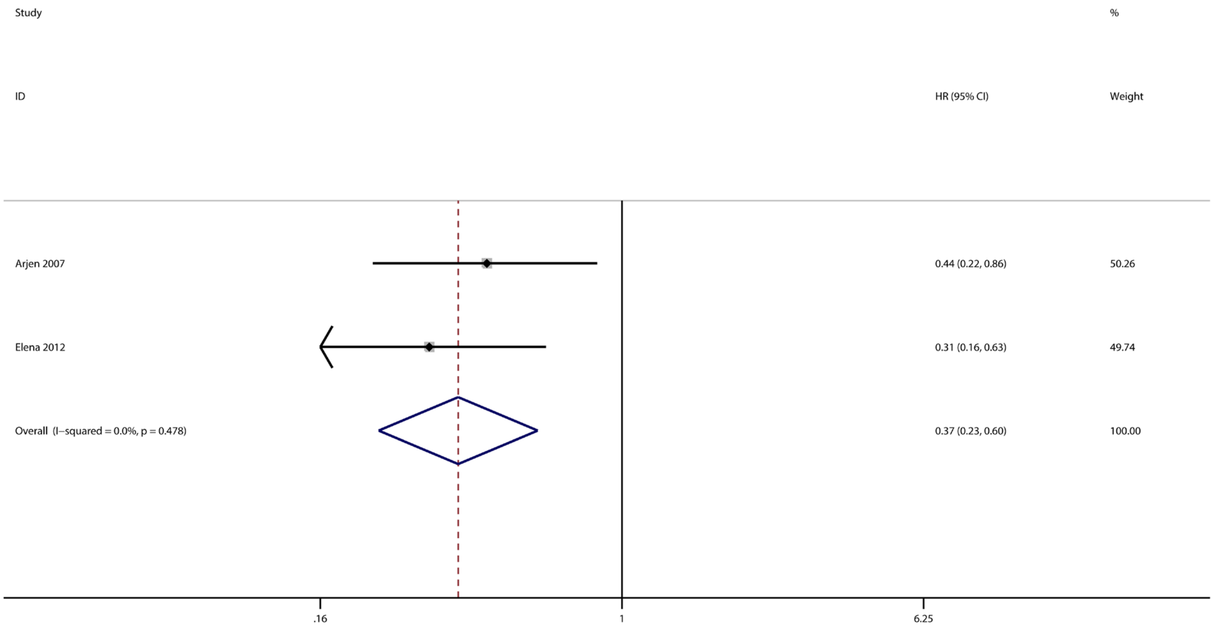

Supplementary Figure 3: Meta-analysis with a random-effect model for the association between GLUT-1 and OS in colorectal cancer.
